# Supplementary material for: Adjuvant Therapy with Budesonide Post-Kasai Reduces the Need for Liver Transplantation in Biliary Atresia
Source: J Clin Med. 2021 Dec 9;10(24):5758. doi: 10.3390/jcm10245758 (PMC8704494; doi:10.3390/jcm10245758)
Supplement: Supplementary file 1 [file jcm-10-05758-s001.zip › jcm-1485001-supplementary.pdf]

Table S1. Demographic and laboratory features of the study population.

	Study group (range)	Control group (range)	p-value
**Gestational age (weeks)**	38,5 (28 – 42)	38,6 (25 – 42)	n. s.
**Male/female (%)**	37%/63%	42%/58%	n. s.
**Syndromatic form (%)**	10%	12%	n. s.
**Age at KPE (days)**	60 (26 – 142)	65 (16 – 150)	n. s.
**AST at KPE (IU/l)**	238 (50 – 1307)	234 (40 – 1059)	n. s.
**ALT at KPE (IU/l)**	162 (25 – 654)	157 (20 – 662)	n. s.
**GGT at KPE (IU/l)**	549 (66 – 2164)	549 (66 – 1470)	n. s.
**Bilirubin at KPE (µmol/l)**	136 (70 – 244)	156 (66 – 347)	p = 0.009

Table S2. Outcome related to the age in days, when the Kasai procedure was performed.

Age at KPE (days)	SOA	SNL	SNLjf
< 31	83% (10/12)	25% (3/12)	17% (2/12)
31-60	91% (68/75)	52% (39/75)	48% (36/75)
> 60	87% (64/74)	37% (27/74)	30% (22/74)

Table S3. Compilation of 14 adjuvant therapy studies after Kasai-portoenterostomy.

Author	Study design and OEBM	Patients/controls	Follow up rate % months	Drug Administration	Outcome parameters	Survival with native liver	Incidence LTx	Conclusion and comments
Meyers (2003) [1]	R IIIb	14/14	96.3% 42 months	(Methyl)prednisolone i.v. and p.o. (1)	SNL (3 -4 m) IncTx (12 m)	improved	reduced	steroids may improve the clearance of jaundice
Kobayashi (2005) [2]	R IIIb	51/12	n.a.	Prednisolone i.v. (2)	n.i.	n. i.	n. i.	not analysable
Escobar (2006) [3]	R IIIb	21/22	88.4% n.i.	Prednisolone i.v. (3)	CoJ (6 m) incLTx	improved	not reduced	significantly improved clearance of jaundice after 6 months
Vejchapipat (2007) [4]	R IIIb	33/20	n.i.	Prednisolone p.o. (4) oral	SNL (6 m)	not improved		no effect
Davenport (2007) [5]	P Ib	36/37	94.4% 12 months	Prednisolone p.o. (5) oral	jfSNL (6m and 12)	not improved	not reduced	initially improved clearance of jaundice
Petersen (2008) [18]	P IIb	49/20	100% 24 – 66 months	(Methyl)prednisolone i.v. p.o. (6)	SNL (6m and 24 m)	not improved	not reduced	high dose steroids do not reduce LTx incidence
Chung (2008) [6]	R IIIb	13/17	n.i.	Prednisolone p.o. (7)	SNL and incLTx (3 and 6 m)	not improved	not reduced	initially improved clearance of jaundice
Davenport (2013) [7]	P Ib	62/91	100% 24 months	Prednisone p.o. (8)	jfSNL (6m) SNL (48 m)	not improved	not reduced	iInitially improved clearance of jaundice

Bezerra (2014) [8]	P, DR, PC Ib	70/70	90.7% 24 months	(Methyl)prednisolone i.v. and p.o. (9)	jfSNL (6 m) SNL (24 m)	not improved	not reduced	High dose steroids do not reduce LTx incidence
Tanaka (2019) [9]	R IV	16/?	n.i.	(Methyl)prednisolone i.v. and p.o. (10)	CoJ 3m	n.i.	n.i.	not analysable
Kumar (2019) [10]	R IV	79/?	87.1% 24 months	Prednisolone p.o. (11)	CoJ (6m)	n.i.	n.i.	not analysable
Parolini (2019) [11]	R IIIb	8/28	100% 6 months	Ganciclovir Valganciclovir i.v. and p.o. (12)	SNL (24 m)	n.i.	n.i.	no clear message
Mack (2019) [12]	P IIb	29/64	96.7% 12 months	Immunoglobulines i.v. (13)	incLTx (12 m)	not improved	not reduced	no improvement of SNL with immunoglobulines
Pietrobattista (2020) [9]	P IIb	25/18	100% 24 months	(Methyl)prednisolone i.v. and p.o. (14)	SNL (24 m)	not improved	not reduced	no effect
This study	R IIb	107/83	100% 6, 12, 24, 122 and 229 months	Budesonide rectally	SNL, jfSNL (6m, 24m, 122 m, 229 m)	improved	reduced	Significantly reduced need for LTx

Study design: R - retrospective; P – prospective; RD – randomized; PC – placebo controlled. OEBM: Oxford Centre for Evidence Based Medicine, available at: http://www.cebm.net. Accessed December 1, 2020. Administration: i.v. – intravenously; p.o. orally; n.i. no information. Outcome parameters: SNL – survival with native liver (months after Kasai); jfSNL – Jaundice free survival with native liver (months after Kasai); incLTx - incidence LTx; CoJ - Clearance of jaundice (not defined). Dosage and application:

(1) Tapering 10, 8, 6, 5, 4, 3, 2 mg/kg/d (1 week), follow by prednisone p.o. 2 mg/kg/d for 8 – 12 weeks; (2) 4 different protocols (P), each dose was given for 3 days: P1: 6, 4, 2mg, P2: 10, 5, 2mg, P3: 20, 15, 10, 5, 2mg, P4: same as P3 + each time stools turn pale, protocol was restarted at 20mg; (3) Doses varied from 2 mg/kg/d (low) to 20 mg/kg/d (pulses) tapering over 2 – 6 weeks; (4) 4 mg/kg/d for 3 to 4 days, then at alternate days for 1 – 3 months; (5) 2mg/kg/d day 7 to 21and 1mg/kg/d day 22 to 28; (6) Methylprednisolone i.v. 10mg/kg/d day 1 to 5 followed by Methylprednisolone p.o. 1mg/kg/d day 26 to 28; (7) Prednisolone p.o. 4mg/kg/d week 2 – 3, 2mg/kg/d week 4 – 5, 1mg/kg/d week 6 – 7; (8) Low dose regimen: Prednisolone p.o. 2mg/kg/d day 7 to 2, 1mg/kg/d day 22 to 28, High dose regimen: Prednisolone p.o., 5mg/kg/d day 5 to 9, 4mg/kg/d day 10 to 14, 3mg/kg/d day 15 to 19, 2mg/kg/d day 20 to 24, 1mg/kg/d day 25 to 29, then Hydrocortisone: 2.5mg/kg twice per dayfrom day 30 to 32 and once per day from day 33 to 35; (9) Methylprednisolone i.v.4mg/kg/d for 2 weeks,followed by prednisolone p.o. 2mg/kg/d for 2 weeks, followed by a 9 week tapering protocol; (10) Prednisolone p.o. 4mg/kg/d for 5 days, 2mg/kg/d for 5 days, 1mg/kg/d for 5 days, 0.5mg/kg/d for 5 days, pulse therapy: methylprednisolone i.v. 20mg/kg/d for 2 days, 10mg/kg/d for 2 days, 5mg/kg/d for 2 days, followed by an oral prednisolone protocol; (11) Prednisolone p.o. 2mg/kg/d for 2 weeks, followed by a 4 week tapering protocol; (12) Ganciclovir i.v. 5mg/kg/d for 1 week, followed by valganciclovir p.o. 520mg/m2/d until negativity of CMV DNA titer; (13) immunoglobulines i.v. 1g/kg IVIg at days 3 to 5, 30 and 60 days after KPE; (14) Methylprednisolone i.v. 10mg/kg/d, decreasing 2mg/kg/d every 24h, until the dose of 2mg/kg/d, followed by prednisolone p.o. 2mg/kg/d for 15 days, 1mg/kg/d for 15 days, 0.5mg/kg/d for 15 days, 0.25mg/kg/d for 15 days

References

1 Meyers RL, Book LS, O'Gorman MA, JacksonWD, Black RE, Johnson DG, et al. High-dose steroids, ursodeoxycholic acid, and chronic intravenous antibiotics improve bile flow after Kasai procedure in infants with biliary atresia. J Pediatr Surg 2003;38:406-11.

2 Kobayashi H, Yamataka A, Koga H, Okazaki T, Tamura T, Urao M, et al. Optimum prednisolone usage in patients with biliary atresia postportoenterostomy. J Pediatr Surg 2005;40:327-30.

3 Escobar MA, Jay CL, Brooks RM, West KW, Rescorla FJ, Molleston JP, et al. Effect of corticosteroid therapy on outcomes in biliary atresia after Kasai portoenterostomy. J Pediatr Surg 2006;41:99-103.
4 Vejchapipat P, Passakonnirin R, Sookpotarom P, Chittmittrapap S, Poovorawan Y. High-dose steroids do not improve early outcome in biliary atresia. J Pediatr Surg 2007;42:2102-5.
5 Davenport M, Stringer MD, Tizzard SA, McClean P, Mieli-Vergani G, Hadzic N. Randomized, double-blind, placebo-controlled trial of corticosteroids after Kasai portoenterostomy for biliary atresia. Hepatology 2007;46:1821-7.
6 Chung HY, Wong KKY, Cheun Leung Lan LCL, Tam PKH. Evaluation of a standardized protocol in the use of steroids after Kasai operation. Pediatr Surg Int 2008;24:1001-4.
7 Davenport M, Parsons C, Tizzard S, Hadzic N. Steroids in biliary atresia: single surgeon, single centre, prospective study. J Hepatol 2013;59:1054-8.
8 Bezerra JA, Spino C, Magee JC, Shneider BL, Rosenthal P, Wang KS. Use of corticosteroids after hepatoportoenterostomy for bile drainage in infants with biliary atresia: the START randomized clinical trial. JAMA 2014;311:1750-9.
9 Tanaka Y, Shirota C, Tainaka T, Sumida W, Oshima K, Makita S, et al. Efficacy of and prognosis after steroid pulse therapy in patients with poor reduction of jaundice after laparoscopic Kasai portoenterostomy. Pediatr Surg Int 2019;35:1059-63.
10 Kumar R, Lal BB, Sood V, Khanna R, Kumar S, Bharathy KGS, et al. Predictors of Successful Kasai Portoenterostomy and Survival with Native Liver at 2 Years in Infants with Biliary Atresia. J Clin Exp Hepatol 2019;9:453-9.
11 Parolini F, Hadzic N, Davenport M. Adjuvant therapy of cytomegalovirus IgM + ve associated biliary atresia: Prima facie evidence of effect. J Pediatr Surg 2019;54:1941-5.
12 Mack CL, Spino C, Alonso EM, Bezerra JA, Moore J, Goodhue C, et al. A Phase I/IIa Trial of Intravenous Immunoglobulin Following Portoenterostomy in Biliary Atresia. J Pediatr Gastroenterol Nutr 2019;68:495-01.

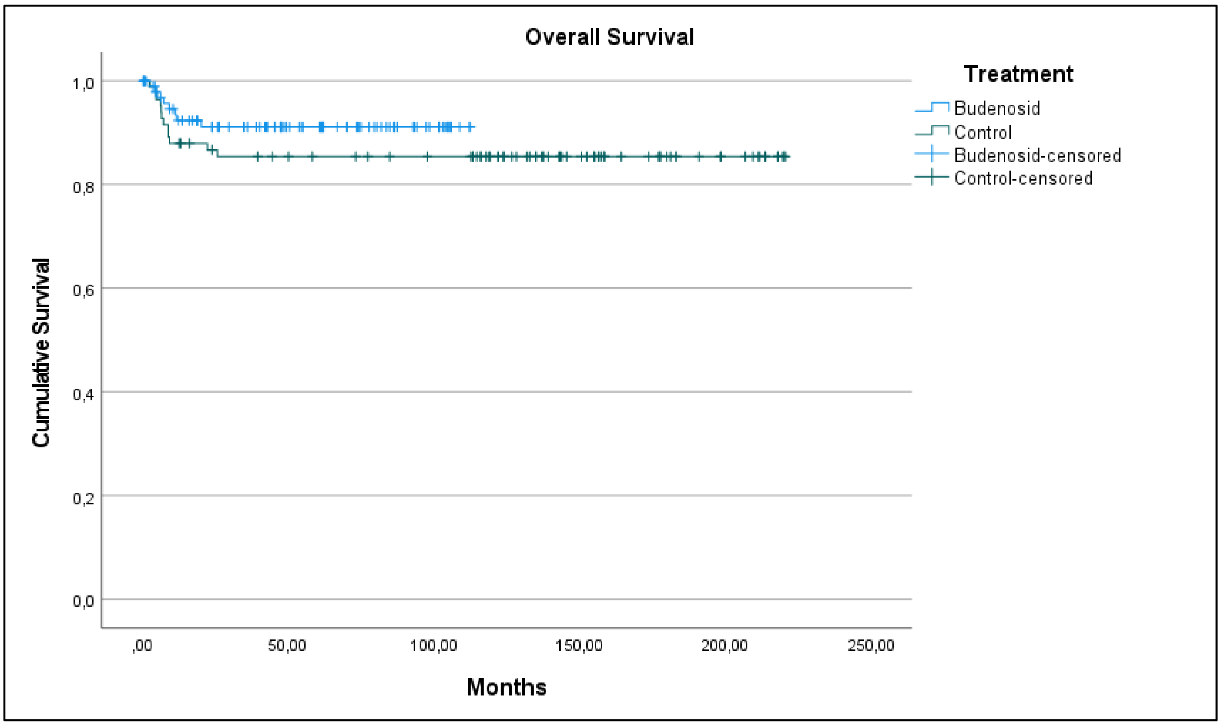

Figure S1. Kaplan-Meier curves: survival over all.
